# Comparison of Disorder-Specific Group CBT and Generic Group CBT in Treating Adolescents with Social Anxiety Disorder: A Randomized Controlled Trial

**DOI:** 10.1007/s10802-025-01412-z

**Published:** 2026-02-06

**Authors:** Thea Nørregaard Agersnap, Johanne Jeppesen Lomholt, Morten Berg Jensen, Mikael Thastum

**Affiliations:** 1 Department of Psychology and Behavioral Sciences, Aarhus BSS, Aarhus University, Bartholins Alle 11, Aarhus C, 8000 Denmark; 2 Department of Economics and Business Economics, Aarhus BSS, Aarhus University, Fuglesangs Allé 4, Aarhus C, 8000 Denmark

**Keywords:** Social anxiety disorder, Adolescents, Cognitive behavioral therapy, Group therapy, Randomized controlled trial

## Abstract

Social anxiety disorder is one of the most common disorders in adolescence. It is associated with severe distress, negative long-term consequences, and chronicity. Cognitive behavioral treatment (CBT) is the recommended treatment for social anxiety disorder. However, adolescents with social anxiety disorder consistently show poorer outcomes than any other anxiety disorder. The aim of the study was to compare the efficacy of a disorder-specific group CBT to a generic group CBT in treating adolescents with social anxiety disorder. The disorder-specific group CBT was specifically enhanced for adolescents with social anxiety disorder, while the generic group CBT was based on traditional CBT methods designed for all types of anxiety disorders. Ninety adolescents aged 12–17 with social anxiety disorder as their primary disorder participated in the randomized controlled trial. Semi-structured diagnostic interviews and questionnaires were completed at baseline, post-treatment, and 3-month follow-up. The disorder-specific group CBT and the generic group CBT showed no significant difference over time. However, both the disorder-specific group CBT and the generic group CBT showed significant improvements in outcomes over time equivalent to medium to large effect sizes. These results indicated that, regardless of treatment condition, the group format exclusively for adolescents with primary social anxiety disorder could be a feasible approach.

Trial registration

ClinicalTrials.gov. Identifier NCT03986827.

**Supplementary Information:**

The online version contains supplementary material available at 10.1007/s10802-025-01412-z.

## Introduction

Cognitive behavioral therapy (CBT) is the best-documented treatment for anxiety disorders in children and adolescents (James et al., 2020). Often CBT treatment for anxiety disorders, including social anxiety disorder, follows a generic approach, where all types of anxiety disorders are treated with the same treatment manuals. These generic CBT treatment manuals target the underlying causes and maintaining factors, that are common across different anxiety disorders, like unrealistic thoughts and avoidance (Evans et al., 2021; Kendall & Hedtke, 2006; Rapee et al., 2019a). Generally, generic CBT treatments have shown solid effects for children and adolescents with anxiety disorders (e.g. Arendt et al., 2016; James et al., 2020; Rapee et al., 2017; Reynolds et al., 2012). Nevertheless, a meta-analysis concludes that adolescents with social anxiety disorder have poorer treatment outcomes, compared to adolescents with other anxiety disorders. They discovered that only 35% of children and adolescents with primary social anxiety disorder were free of their primary disorder post-treatment, compared to 54% of those with other primary anxiety disorders. Additionally, the findings revealed that children and adolescents with social anxiety disorder were 48% less likely to recover compared to those with any other primary anxiety disorder (Evans et al., 2021). The reasons for these poorer treatment outcomes from CBT for adolescents with social anxiety disorder, compared to other anxiety disorders, are not yet clear.

Social anxiety disorder is one of the most common disorders in adolescence, with a prevalence of 5–9% among 13–18 years of age (Burstein et al., 2011; Costello, Egger & Angold, 2005; Kessler et al., 2012). Untreated social anxiety disorder can lead to a range of negative outcomes, including chronicity, problems in school, loneliness, increased risk of other anxiety disorders, depression, and substance abuse (Beesdo-Baum et al., 2012; Beesdo et al., 2007; Burstein et al., 2011; Kim-Cohen et al., 2003; Roza et al., 2003). Adolescence seems to be a critical period for treatment of social anxiety disorder to prevent a chronic developmental course (NICE, 2013; Spence & Rapee, 2016). Therefore, it seems highly relevant to investigate treatment options for adolescents with social anxiety disorder, to hopefully improve treatment outcomes.

For adults with social anxiety disorder, the National Institute for Health and Care Excellence (NICE, 2013) recommends individual disorder-specific CBT based on either Clark and Wells (1995) or Heimberg models (e.g. Hope et al., 2006) of social anxiety disorder. Where the Clark and Wells (1995) model highlights several cognitive processes in maintaining social anxiety disorder, including negative assumptions, self-focused attention, safety behaviors, and post-event processing, Heimberg’s model builds upon this cognitive framework, but also integrates behavioral elements, such as avoidance behaviors. A network meta-analysis of adults with social anxiety disorder found that individual disorder-specific CBT based on Clark and Wells (1995) model showed the most consistent evidence of greater effects (Mayo-Wilson et al., 2014). Their suggestion was drawn because the 95% credible intervals for individual disorder-specific CBT based on Clark and Wells model (1995) did not overlap with most other psychological interventions. Moreover, this approach showed the highest standardized mean difference, compared to other psychological treatments and pharmacological treatments (Mayo-Wilson et al., 2014). Disorder-specific CBT based on Clark and Wells (1995) model is specifically enhanced to treat social anxiety disorder. It incorporates techniques designed to target maintaining processes of social anxiety disorder, such as safety behavior, self-focused attention, and post-event processing. Leigh and Clark (2018) conducted a review of the literature and found evidence indicating that the maintaining factors proposed in Clark and Wells (1995) model were also applicable to adolescents with social anxiety disorder. This makes it plausible that disorder-specific CBT could enhance treatment outcomes for adolescents (Leigh & Clark, 2018; Spence & Rapee, 2016). This aligns with findings from a meta-analysis by Reynolds et al. (2012) that indicate that disorder-specific CBT treatment could improve outcomes for adolescents with social anxiety disorder. Reynolds et al. (2012) included 55 studies of treatment for anxiety disorder in children and adolescents. They included both generic CBT approaches, that apply the same treatment techniques for different anxiety disorders, as well as disorder-specific CBT approaches, that were enhanced with treatment techniques specifically designed to treat a particular type of anxiety disorder. Among these, nine studies focused on disorder-specific CBT for social anxiety disorder, and they demonstrated larger effect size compared to generic CBT in the meta-analysis. However, it is important to note that the meta-analysis did not directly compare generic and disorder-specific CBT treatments for social anxiety disorder.

To the best of our knowledge, only a limited number of studies have directly compared disorder-specific CBT to generic CBT for social anxiety disorder in children and adolescents. Simultaneously with the study by Rapee et al. (2023), we conducted the current study with the same objective, which was to compare disorder-specific CBT to generic CBT using the same evidence-based manualized Cool Kids Anxiety Program (Rapee et al., 2019a) as the foundation for both treatment conditions. However, while Rapee et al. (2023) implemented their intervention in an individual format, we tested the treatment conditions in a group format as presented in this paper.

In both studies the generic CBT was based on standard CBT techniques from the evidence-based manualized Cool Kids Anxiety Program (Rapee et al., 2019a). The disorder-specific CBT in both studies incorporated additional treatment techniques to the standard Cool Kids Anxiety Program, based on Clark and Wells’ (1995) model for social anxiety disorder. These techniques included reducing safety behaviors and post-event processing, training attention to reduce self-focused attention, emphasizing consequences in cognitive restructuring and (in-vivo) exposure, and using feedback experiments to build a more realistic self-concept.

Despite the methodological similarities, the study by Rapee et al. (2023) differs from the current study in several factors. Rapee et al. (2023) included children and adolescents aged 7–17 with social anxiety disorder, the participants had an anxiety disorder as their primary diagnosis, though their primary anxiety diagnosis was not necessarily social anxiety disorder, and excluded participants with frequent school nonattendance. In contrast, the current study focused specifically on adolescents aged 12–17 years with primary social anxiety disorder and did not exclude school absenteeism. Additionally, whereas Rapee et al. (2023) tested the treatments in an individual format, we tested a group format consisting of groups with four adolescents with primary social anxiety disorder. A group format may facilitate more frequent social exposure, promote peer interaction, and be more cost-effective (Yang et al., 2019). Moreover, a meta-analysis found comparable treatment effects for individual (Hedges’ g = 1.10) and group format (Hedges’ g = 1.19) in psychological treatments for social anxiety disorder (Yang et al., 2019), and NICE guidelines (2013) recommend both formats for adolescents with social anxiety disorder. To our knowledge, no prior study has directly compared disorder-specific and generic CBT in a group setting.

Rapee et al. (2023) have published the results of their study, reporting no significant differences between individual disorder-specific CBT and individual generic CBT. However, they observed a trend suggesting a potential advantage of disorder-specific CBT in terms of remission from social anxiety disorder at 6-month follow-up. Previous research has mainly focused on individual CBT. Spence, Donocan, March, Kenardy, and Hearn (2017) compared disorder-specific and generic CBT in an online format for children and adolescents aged 8–17 with a primary social anxiety disorder and found no significant differences in outcomes. In contrast, Ingul et al. (2014) reported superior outcomes for individual disorder-specific CBT compared to generic group CBT for adolescents aged 13–16 with a primary social anxiety disorder.

Given the lack of direct comparisons between disorder-specific and generic CBT in a group format, the present study aimed to address this gap. We sought to compare the efficacy of disorder-specific group CBT to generic group CBT in adolescents with primary social anxiety disorder. The disorder-specific group CBT was adapted based on Clark and Wells’ (1995) model, while the generic group CBT followed traditional CBT principles applicable to all anxiety disorders. We hypothesized that both treatments would reduce social anxiety symptoms, with disorder-specific group CBT leading to greater reductions compared to generic group CBT.

## Methods

The current study was pre-registered in Clinical Trials, identifier: NCT03986827. The article follows the CONSORT guidelines for randomized controlled trials. The study has obtained approval from the Institutional Review Board at Aarhus University (j.nr. 2019-616-000013) and the Institutional Data Protection Agency at Aarhus University (j.nr. 2016-051-000001).

### Study Design

The study was a single-site randomized controlled superiority design of disorder-specific group CBT compared to generic group CBT treating social anxiety disorder among adolescents aged 12–17. The design was a two (disorder-specific group CBT and generic group CBT) by three (pre, post, 3-month follow-up) mixed between-within design.

### Randomization and Blinding

Around four treatment groups started every half year. Randomization was conducted every half year before the next round of treatment started. Participants were randomized in a balanced 1:1 ratio to the two treatment conditions. One exception was made to the 1:1 ratio randomization, due to recruitment problems during the first national COVID-19 lockdown. During this lockdown, we could only recruit participants to three treatment groups, explaining the lack of 1:1 ratio randomization that one time. The randomization was conducted by an external secretary using an online computer generator (www.random.org). All participants were blinded to their treatment condition and were only informed that they had been randomly assigned to one of two types of CBT treatments. The interviewers who conducted the Anxiety Disorder Interview Schedule-IV C/P (ADIS-IV C/P) interviews were blinded to which treatment conditions the participants should receive or had received.

### Procedure

The research was conducted at the Centre for Psychological Treatment of Children and Adolescents (CEBU), a teaching and research facility at the Department of Psychology and Behavioral Sciences at Aarhus University in Denmark. Information about the treatment was disseminated through various channels, including newspapers, websites, social media, and local schools. Participants were self-referred via CEBU’s website, where their parents provided brief descriptions of their adolescent’s problems. Following an initial screening, potential participants completed a semi-structured diagnostic interview (ADIS-IV C/P). The interviews were conducted face-to-face by clinical psychologists or graduate psychology students trained in conducting the interviews. All interviews were recorded on video. However, due to the COVID-19 pandemic, and subsequent national lockdowns, some interviews were conducted online. If the participants were found eligible, they were randomized into one of the two treatment conditions and they completed the questionnaires. The interviews and questionnaires were further assessed after the treatment (post), and three months following the treatment (3-month follow-up). Whenever possible, the interviews were conducted immediately after the families participated in session 10 (post) and again immediately after the booster session (three-month follow-up). The questionnaires were sent out the same day, and the average days from pre- to post-treatment assessment were 128 days (SD = 22.5) and 204 days (SD = 15.3) from pre-treatment to three-month follow-up. The recruitment period overlapped with parts of the timeframe in Rapee et al. (2023). Recruitment began in January 2019 and concluded in September 2021, with all follow-up data available by April 2022.

### Participants

A total of 119 participants were deemed eligible after the initial screening of their brief descriptions, and they completed the interview and the questionnaires. Of those, 90 participants were included in the study. No incentives were offered to participants for their participation in the treatment. Inclusion criteria were adolescents aged 12–17 who met the criteria for a social anxiety disorder as their **primary diagnosis** (determined through the ADIS-IV C/P interview). The exclusion criteria were as follows: autism spectrum disorder, untreated ADHD/ADD, eating disorders, psychotic symptoms, severe self-harm or suicidal ideation, clinical severity rating exceeding 6 on depression (assessed through the ADIS-IV C/P interview), and former CBT for anxiety disorder within the past two years.

**Fig. 1:**
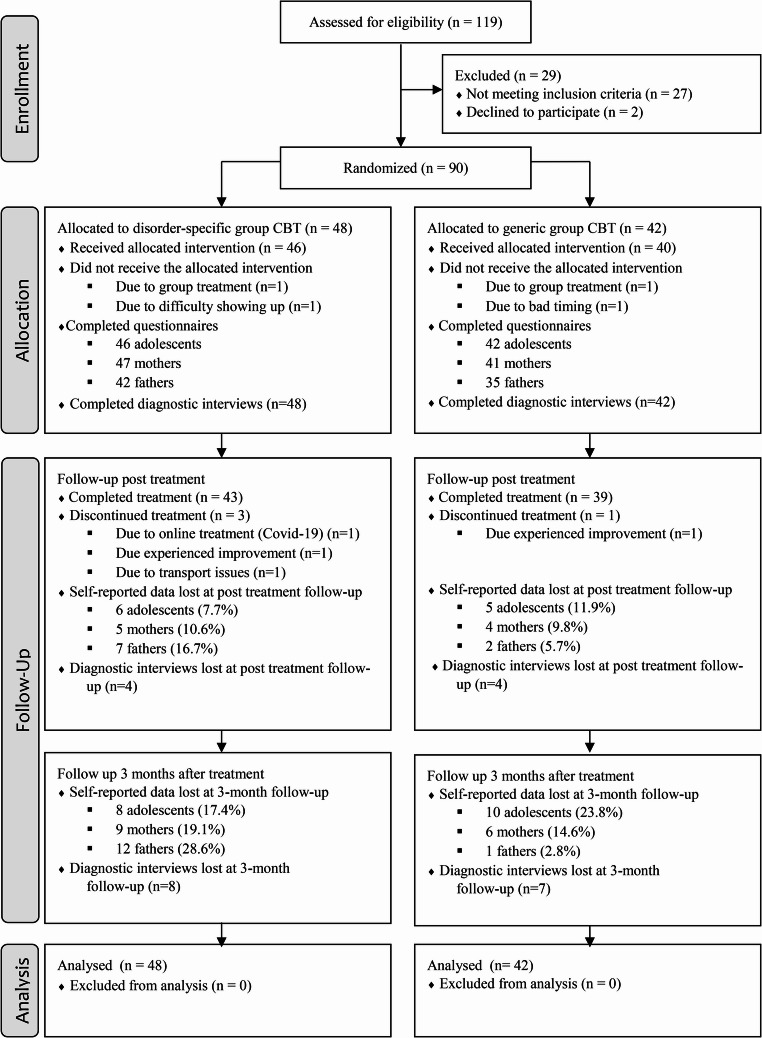
CONSORT flowchart

Ninety adolescents and their parents were enrolled in the study and randomized to either the disorder-specific group CBT (*n* = 48) or the generic group CBT (*n* = 42) (see Fig. 1). The mean age of the participants was 14.6 years (1.4 SD) with 73 individuals (81.1%) identified as female. Additional baseline information is presented in Table 2.

### Interventions

The two treatment conditions were identical to those in Rapee et al. (2023) study but were translated into Danish and adapted to a group format by the staff at CEBU. Both treatment conditions were based on the Cool Kids Anxiety Program (Rapee et al., 2019a), a semi-structured manualized, and empirically validated generic CBT treatment for children and adolescents suffering from all types of anxiety disorders. The treatment manuals for both conditions were developed at Macquarie University in Sydney, Australia.

#### The generic CBT

manual was developed to address all types of anxiety disorders, however, in the current study, only adolescents with a primary social anxiety disorder participated. The generic group CBT closely aligned with the traditional Cool Kids Anxiety Program (Rapee et al., 2019a), which utilizes standard CBT techniques to treat all types of anxiety disorders. These techniques included psychoeducation, setting goals, rewards, parent behavior, cognitive restructuring, exposure (as both homework and in-vivo exposure), calm breathing, problem-solving, and homework (see Table 1). Therapists delivering the generic group CBT were explicitly instructed not to use the additional techniques from the disorder-specific group CBT.

**Table 1:** Overview of the treatment sessions in both treatment conditions

Session number	Participants	Session content for disorder-specific group CBT	Session content for generic group CBT
1	T, A, P	• Psychoeducation about social anxiety	• Psychoeducation about social anxiety
		• Setting goals	• Setting goals
		• Worry scale	• Worry scale
		• Linking thoughts and feelings	• Linking thoughts and feelings
2	T, A, P	• Introduce cognitive restructuring	• Introduce cognitive restructuring
		• Introduce attention training	
3	T, A, P	• Cognitive restructuring	• Cognitive restructuring
		• Introduce rewards	• Introduce rewards
		• Introduce cognitive restructuring to consequences	• Introduce calm breathing
		• Introduce cognitive restructuring *after* a situation	
4	T, A, P	• Introduce exposure (behavioral experiments)	• Introduce exposure (graded exposure)
5	T, A, P	• Parent behavior	• Parent behavior
		• Introduce exposure to consequences	• Introduce simplified cognitive restructuring (in my mind and with notepads)
6	T, A, P	• Reviewing and planning exposure	• Reviewing and planning exposure
		• Introduce safety behavior	
		• Attention training to reduce self-focused attention	
7	T, A, P	• Reviewing and planning exposure	• Reviewing and planning exposure
		• In vivo exposure	• In vivo exposure
		• Feedback experiments	• Introduce problem-solving
8	T, A, P	• In vivo exposure	• In vivo exposure
9	T, A, P	• Reviewing and planning exposure	• Reviewing and planning exposure
		• In vivo exposure	• In vivo exposure
10	T, A, P	• Review goals	• Review goals
		• Maintenance of goals/setbacks	• Maintenance of goals/setbacks
		• Future plans	• Future plans
Booster	T, A, P	• Focusing on maintaining progress	• Focusing on maintaining progress

*T* therapist, *A* adolescents, *P* parents

#### The disorder-specific CBT

manual was based on Cool Kids Anxiety Program (Rapee et al., 2019a) as well, but enhanced with additional treatment techniques, that targeted mechanisms specifically related to social anxiety disorder, based on Clark and Wells’ (1995) model. This model proposed that social anxiety disorder was maintained by mechanisms such as a negatively biased self-concept, safety behaviors often leading to poor social performance, self-focused attention, and post-event processing. Therefore, the additional techniques in the disorder-specific group CBT included exposure in the form of behavioral experiments aimed at reducing avoidance and safety behaviors, for example by improving eye contact, speaking in a higher tone, or reducing reliance on make-up. Moreover, feedback experiments were conducted to build a more realistic self-concept. For example, the adolescents were asked to present a personal interest to the therapist. Before the experiment, the adolescents rated their expected behavior, such as how much they would blush or stutter. Afterward, the adolescent received feedback from the therapist, or if they agreed to be recorded, from a video recording of their presentation. They learned cognitive restructuring that focused on overestimating probability and consequences of negative evaluations. Cognitive restructuring was used both before and after social situations to reduce anxious thoughts as well as post-event processing. Moreover, an additional technique was training of attention to reduce self-focused attention. Some of the techniques mentioned, such as addressing safety behavior and focusing on overestimating of consequences in cognitive restructuring, are also common techniques in traditional generic CBT treatment for all types of anxiety disorders. However, these techniques were only included in the disorder-specific group CBT, as theory predicted they were central to maintaining social anxiety disorder (Clark & Wells, 1995). Additionally, the disorder-specific group CBT also included some of the same techniques as the generic group CBT, such as psychoeducation, setting goals, rewards, parent behavior, and homework (see Table 1). Therapists delivering the disorder-specific group CBT were explicitly instructed not to use techniques only meant for the generic group CBT (which included calm breathing and problem-solving).

The treatment sessions in both treatment conditions were led by a psychologist and three assisting graduate psychology students. They were trained in both treatment manuals through a one-day workshop and a one-day brush-up half a year later. Both the workshop and the brush-up covered the same content, including all treatment techniques. However, for the time of the brush-up, the therapists had acquired greater experience and familiarity with the treatment manuals and techniques. The psychologists switched between the two treatment conditions every year or half-year. This approach was intended to minimize the bias of therapists’ effects, and to enhance the therapists’ ability to clearly distinguish and adhere to the specific techniques in each treatment condition. However, this approach also carries potential disadvantages. For example, therapists may unintentionally (or in some cases intentionally) incorporate elements from one treatment condition to the other. All psychologists participated in weekly internal supervision (with their colleagues) and external supervision (with an expert in the field) every third week.

Both treatment conditions consisted of 10 two-hour sessions completed within 12–13 weeks and a booster session at 3-month follow-up. Treatment was conducted in groups of approximately four adolescents, with group sizes ranging from three to five adolescents. The optimal size of four was chosen to ensure a sufficient number of participants for group dynamics while avoiding overly large groups, which could potentially increase anxiety levels for adolescents with social anxiety disorder. The parents participated in the treatment sessions as well, as this is the most common practice when using the Cool Kids manual. The adolescents, parents, and therapist were together at the start and end of each treatment session. During the middle of the treatment sessions, the therapist spent around an hour with the adolescents in the group, and afterward, the therapist spent around half an hour with the parents. The adolescents, the parents, and the therapist gathered again at the end of the session for a brief recap of the session, as well as a repetition of the homework to be completed before the next session. All sessions were recorded on video. Due to the COVID-19 pandemic and national lockdowns, three groups underwent adaptions, with four of their treatment sessions being conducted online.

In both conditions, if the families spontaneously mentioned techniques or terms from the other treatment condition, the therapists were allowed to acknowledge the issues but not delve deeper into the topic or present additional information. For instance, if a family in the generic group CBT mentioned that their adolescent spoke softly and avoided eye contact during social interactions, the therapist was allowed to recognize the problem but not introduce the term and techniques related to safety behavior any further.

#### Adherence and Competence 

Adherence and competence were assessed using the Competence and Adherence Scale for Cognitive Behavioral Therapy (CAS-CBT; Bjaastad et al., 2016) in 42 randomly selected video recordings from all 21 treatment groups, representing 18% of the total 231 recorded videos. The CAS-CBT was designed to assess CBT treatments for anxiety disorders in children and adolescents. It comprised 11 items rated on a 7-point Likert scale (0–6), covering homework, process and structure, parental involvement, positive reinforcement, collaboration, flexibility, and session goals. External scoring indicated good adherence and competence in both groups with no differences between treatment conditions. The disorder-specific group CBT had a mean adherence score of 5.36 (0.45 SD) out of 6 and a competence score of 5.28 (0.46 SD) out of 6. Similarly, the generic group CBT had a mean adherence score of 5.61 (0.29 SD) out of 6 and a competence score of 5.44 (0.40 SD) out of 6. There was no significant difference between treatment conditions in either adherence (t(19) = −1.46, *p* = .161) or competence (t(19) = − 0.81, *p* = .430). Focusing on the items measuring specific session goals, the disorder-specific group CBT had a mean adherence score of 5.58 (0.63 SD) and a competence score of 5.36 (0.64 SD), while the generic group CBT had a mean adherence score of 5.65 (0.36 SD) and a competence score of 5.60 (0.46 SD). Again, these differences were not significant for either adherence to specific session goals (t(19) = 0.29, *p* = .773) or competence related to specific session goals (t(19) = − 0.97, *p* = .346).

Two methods were employed to determine potential fidelity breaches. The external reviewers assessed visible breaches in the randomly selected videos, which showed a total of four breaches, where the generic group CBT used techniques that were meant to be used exclusively in the disorder-specific group CBT. The external reviewers were graduate psychology students who had been trained in both the CAS-CBT assessment and the two treatment conditions. They were not involved in any aspects of the data analysis or interpretation of the results in the current study.

Additionally, therapists systematically registered any fidelity breaches following each session. In the generic group CBT 13 breaches occurred across 110 sessions. These breaches included three instances of working with safety behavior, three instances of focusing on consequences in cognitive restructuring, two instances of attempting to reduce self-focused attention, two instances of using exposure to consequences, and three instances of using cognitive restructuring after a situation. In the disorder-specific group CBT, two breaches were reported across 121 sessions: one instance of calm breathing and one instance of simplified cognitive restructuring. Moreover, the therapists’ registrations showed that during both disorder-specific group and generic group sessions, interventions were made six times in each group to address other anxiety disorder problems or other non-anxiety-related problems, for example, incorporating activities within sessions to enhance mood and alleviate depressive symptoms. Nine of 12 instances were addressing depression or depressive symptoms disclosed by the adolescents.

### Outcome Measures 

#### Primary Outcome Measures

##### Anxiety Disorders Interview Schedule for DSM-IV, Child and Parent Version (ADIS-IV C/P; Silverman& Nelles, 1988)

ADIS-IV C/P, a semi-structured diagnostic interview, was utilized to assess anxiety disorders in adolescents following the Diagnostic and Statistical Manual of Mental Disorders (DSM)-IV. The interview also assessed common comorbid disorders (e.g. depression and ADHD) as well as screened for other disorders (e.g. autism spectrum disorder and eating disorders). The interview was conducted separately with the adolescents and the parents, who independently rated symptoms and interference levels. The interview was conducted by clinical psychologists or graduate students in clinical psychology who had been trained to administer the interviews. The interviewers were blinded to the participants’ treatment conditions during the assessments. Following the interview, the interviewers used a 9-point Likert scale ranging from “not disturbed at all” to “severely disturbed” (0–8) to assign each diagnosis, based on the responses, symptoms, and interference ratings reported separately by the adolescents and their parents. CSR scores of 4 or higher indicated a clinical diagnosis. The most severe diagnosis was considered the primary diagnosis. Eighteen videos (20%) of the baseline ADIS-IV C/P interviews were reviewed and recoded by external scorers who were trained in administrating and scoring the ADIS-IV C/P interview. The interrater reliability showed a substantial agreement (*κ* = 0.67). The anxiety section of ADIS-C has demonstrated good validity and test-retest reliability (Silverman et al., 2001; Wood et al., 2002).

##### Social Phobia Inventory (SPIN; Connor et al., 2000)

SPIN measured the adolescents’ social anxiety symptoms, and higher scores indicated greater distress. The adolescents completed the 17-item questionnaire, which consisted of three subscales (fear, avoidance, and physical/bodily reactions), and was measured on a 5-point Likert scale (0–4). SPIN has demonstrated good psychometric properties for assessing adolescents with social anxiety disorder and good internal consistency, test-retest reliability, and convergent and divergent validity (Connor et al., 2000; Ranta et al., 2007a, b). In the current study, SPIN demonstrated good internal consistency at baseline, post-treatment, and three-month follow-up, ranging from α = 0.86–0.93.86.93.

##### Spence Children’s Anxiety Scale (SCAS; Spence, 1998)

SCAS was used to measure the adolescents’ overall anxiety symptoms. Higher scores indicated higher levels of anxiety. SCAS was rated on a 4-point Likert scale (0–3) by both adolescents and parents. It consisted of six subscales reflecting symptoms of social anxiety disorder, separation anxiety disorder, generalized anxiety disorder, panic disorder, agoraphobia, obsessive-compulsive disorder, and fear of physical injury. The Danish version of SCAS has demonstrated good test-retest reliability and good internal consistency in clinical and non-clinical samples (Arendt et al., 2014). In the current study, SCAS demonstrated good internal consistency at baseline, post-treatment, and three-month follow-up, ranging from; α =0.86–0.88.86.88 for adolescents, α =0.90–0.93.90.93 for mothers, and α = 0.92–0.94.92.94 for fathers.

#### Secondary Outcome Measure

##### Child Anxiety Life Interference (CALIS; Lyneham et al., 2013)

CALIS assessed the impact of anxiety symptoms on adolescents’ daily life interferences. It was rated on a 5-point Likert scale (0–4) by both adolescents and parents. Higher scores indicated higher life interference. CALIS has demonstrated moderate test-retest reliability and satisfactory internal consistency (Lyneham et al., 2013). In the current study, CALIS demonstrated acceptable internal consistency at baseline, post-treatment, and three-month follow-up, ranging from; α = 0.75–0.87.75.87 for adolescents, α = 0.88–0.93.88.93 for mothers, and α = 0.91–0.93.91.93 for fathers.

##### Short version of the Mood and Feelings Questionnaire (S-MFQ; Daviss et al., 2006)

The questionnaire measured adolescents’ depressive symptoms in the past two weeks. It consisted of 13 items, rated on a 3-point Likert scale (0–2) by both adolescents and parents. Higher scores indicated higher levels of depressive symptoms. The Danish version of the long version of the MFQ has demonstrated good internal consistency and test-retest reliability (Daviss et al., 2006). In the current study, S-MFQ demonstrated good internal consistency at baseline, post-treatment, and three-month follow-up, ranging from; α = 0.88–0.92.88.92 for adolescents, α = 0.88–0.91.88.91 for mothers, and α =0.89–0.93.89.93 for fathers.

##### Negative Effects Questionnaire (NEQ; Rozental et al., 2019)

NEQ measured negative effects after the psychological treatment and was completed by adolescents and their parents. NEQ was slightly adjusted from the original version to suit the adolescents’ age. The questionnaire inquired about the presence of negative effects during treatment (yes/no), and, if applicable, how negatively the effect was on a 5-point Likert scale (0–4), and finally, whether they attributed the negative effects to “the treatment they received” and/or “other circumstances”. NEQ exhibited acceptable psychometric properties (Rozental et al., 2019). In the current study, NEQ demonstrated acceptable internal consistency at baseline, post-treatment, and three-month follow-up; α = 0.80 for adolescents, α = 0.79 for mothers, and α = 0.89 for fathers.

##### Depression Anxiety Stress Scale (DASS; Lovibond & Lovibond, 1995)

DASS was utilized to measure the symptoms of anxiety, depression, and stress in the parents. It was included since parent pathology was found associated with poorer response for their children (Hudson et al., 2015). DASS consisted of 42 items, which was rated on a 4-point Likert scale (0–3). Higher scores indicated higher distress. DASS has demonstrated good psychometric properties (Antony et al., 1998). In the current study, DASS showed excellent internal consistency at baseline, post-treatment, and three-month follow-up, ranging from; α = 0.95-0.95.95.95 for mothers and α =0.95–0.99.95.99 for fathers.

##### Children’s Automatic Thoughts Scale (CATS, Schniering & Rapee, 2002)

CATS was completed by the adolescents to assess their negative self-statements, which aligned with maintaining factors outlined in Clark and Wells (1995) model of social anxiety disorder. The full questionnaire consisted of four subscales related to automatic thoughts on personal failure, social threats, physical failure, and hostility. The personal failure and social threats subscales were included in the current study. They consisted of 20 items rated on a 5-point Likert scale (0–4). These subscales correlated with self-reported social anxiety in another study (Micco & Ehrenreich, 2009). CATS has demonstrated good psychometric properties (Micco & Ehrenreich, 2009; Schniering & Lyneham, 2007; Schniering & Rapee, 2002). In the current study, CATS indicated excellent internal consistency at baseline, post-treatment, and three-month follow-up, ranging from α = 0.95–0.97.95.97.

##### Subtle Avoidance Frequency Examination (SAFE, Cuming et al., 2009)

SAFE measured the adolescents’ safety behaviors, including direct and indirect safety behaviors, as well as safety behaviors aimed at avoiding or concealing physical symptoms. SAFE consisted of 32 items rated on a 5-point Likert scale (0–4). SAFE has demonstrated good psychometric properties for adolescents (Cuming et al., 2009; Thomas et al., 2012). In the current study, SAFE indicated good internal consistency at baseline, post-treatment, and three-month follow-up, ranging from; α = 0.89–0.93.89.93.

##### Experience of Service Questionnaire (ESQ)

ESQ measured the adolescents and parents’ satisfaction with the treatment. The questionnaire was adjusted from the original ESQ version (Attride-Stirling, 2002). ESQ for the adolescents contained 7 items, and the parent version contained 10 items, each rated on a 3-point Likert scale (0–2). The questionnaire provided an opportunity for qualitative feedback. In the current study, internal consistency for ESQ ranged from questionable to good at post-treatment for adolescents (α = 0.73), mothers (α = 0.68), and fathers (α = 0.87).

#### Background Information

The parents provided background information, including details about adolescents’ and parents’ mental and physical health, parents’ education level, household income, school absenteeism, and adolescents’ prior or ongoing treatment.

### Statistical Methods

A statistical power analysis revealed that 47 participants in each treatment condition yielded an acceptable power of 0.81 (α = 0.05, two-tailed) (Lassen et al., 2019). This was based on an estimated effect size; *d* = 0.65. The effect size was estimated based on previous similar studies that found medium to large effect sizes (Arendt et al., 2016; Ingul et al., 2014).

Stata version 18 was used for all analyses. Descriptive statistics (mean, standard deviation, and percentages) were used to describe the sample at baseline, post-treatment, and 3-month follow-up. T-test and chi^2^ were used to analyze differences in baseline characteristics as presented in Table 2. Linear mixed models based on the intention-to-treat principle were used to compare groups over time for all continuous outcome measures. This was done by determining the time*group interaction effect as well as the effect over time. All models included a random intercept. A random slope was specified if it improved the fit of the model (evaluated by a significant change in −2 log-likelihood value (Heck et al., 2014). Due to variation in days from pre- to post- to follow-up assessment the time variable was calculated as the number of days from each participant’s baseline assessment. In addition to a linear specification of time, various nonlinear transformations (logarithmic, exponential, quadratic) were also investigated. There were no discernible differences in the log-likelihood value and therefore the linear specification was maintained. A nonlinear mixed model was used to determine change over time for the ADIS-IV C/P social anxiety diagnosis. The multilevel analysis (using Maximum Likelihood) accounted for missing values in the dependent variable as an integral part of the analysis.

**Table 2:** Baseline characteristics for both treatment conditions

	Disorder-specific (*n* = 48)	Generic (*n* = 42)
Age, mean (SD)	14.4 (1.4)	14.8 (1.5)
Gender (girls), no. (%)	35 (72.9)*	38 (90.5)*
Living with both parents, no. (%)	31 (66)	25 (61)
Children receive medication for anxiety disorder, no. (%)	0 (0)	3 (7.1)
Education level for mothers/fathers		
Further and higher education, no. (%)	12 (25.5)/15 (35.8)	14 (34.1)/10 (28.6)
Vocational education, no. (%)	20 (42.6)/15 (35.8)	22 (53.7)/10 (28.6)
Elementary or high school equivalent, no. (%)	15 (31.9)/12 (28.6)	5 (12.2)/15 (42.9)
Number of school days missed in the last month		
More than 7 days, no. (%)	10 (4.2)	16 (38.1)
3–6 days, no. (%)	8 (16.7)	4 (9.5)
1–2 days, no. (%)	7 (14.6)	6 (14.3)
None, no. (%)	20 (41.7)	11 (26.2)
Moms with mental illness, no. (%)	10 (20.8)	7 (16.7)
Dads with mental illness, no. (%)	4 (8.3)	4 (9.5)
ADIS-IV C/P CSR on social anxiety disorder, mean (SD)	6.3 (1.0)	6.3 (1.0)
Comorbid anxiety disorder, no. (%)	28 (58.3)	26 (61.9)
Comorbid depression (or dysthymia), no. (%)	11 (22.9)	14 (33.3)
Number of comorbid anxiety disorders, mean (SD)	1.0 (1.1)	1.3 (1.3)
Number of other comorbid disorders, mean (SD)	0.3 (0.6)	0.4 (0.5)

Note: *indicating a significant difference between disorder-specific CBT and generic CBT: *x*^2^(1) = 4.5, *p* =.034

Effect sizes were calculated for all outcome measures, calculated from the formula: *d* = 2 × √ (t^2^/df), with t and degrees of freedom (df) based on the Kenward and Roger (1997) approximation. In the context of Cohen’s *d* (Cohen, 1988), effect sizes of 0.2, 0.5, and 0.8 are typically considered as indicating small, medium, and large effect sizes, respectively.

To account for multiple comparisons, we applied a Bonferroni correction to the p-value for our primary outcome measures (ADIS-IV C/P, SPIN, SCAS adolescent, SCAS mothers, and SCAS fathers). Since all pairwise combinations of these five variables were compared, this resulted in a total of 10 pairwise comparisons. Accordingly, the significance level was adjusted to control for Type I error (α = 0.05/10), resulting in an adjusted two-tailed significance level of *α* < 0.005. Our secondary outcome measures were considered exploratory, and accordingly, their significance levels were not adjusted.

## Results

### Baseline Characteristics

Ninety adolescents aged 12–17 (mean = 14.6, SD = 1.4) with social anxiety disorder as their primary disorder participated in treatment. Fifty-four (60%) had a comorbid anxiety disorder. Seventy-three (81.1%) participants identified as girls, Of the 17 boys (18.9%) in the study, four were included in the generic group CBT and 13 were included in the disorder-specific group CBT, resulting in a significant difference in gender distribution between treatment conditions. Additional information on the participants’ baseline characteristics is presented in Table 2.

### Missing Data

As shown in Fig. 1, post-treatment complete data were missing for 13 (14.4%) adolescents, 9 (10%) mothers, and 20 (22.2%) fathers. At 3-month follow-up, complete data were missing for 17 (18.9%) adolescents, 17 (18.9%) mothers, and 26 (28.9%) fathers. Little’s MCAR test confirmed data were missing completely at random at post-treatment, χ²(29) = 35.91, *p* =.176, and follow-up, χ²(24) = 27.67, *p* =.274.

### Primary Outcome Measures

The primary outcomes measured social anxiety disorder, severity of social anxiety symptoms, and overall anxiety symptoms. Outcome measures are reported in Table 3.

**Table 3:** Mean, standard deviations, and main effects for all outcome measures

Measure	Condition	Baseline	Post treatment	3-month follow-up	Time	Interaction effect (Time*condition)
		**M (SD)**	**M (SD)**	**M (SD)**	**β**, ***p*** **[95% CI] (Cohen’s d)**	**β**, ***p*** **[95% CI] (Cohen’s d)**
SPIN	Disorder-specific	35.59 (10.95)	27.4 (12.76)	21.76 (14.11)	− 0.09, *0.000* [−0.11;−0.06] (1.81)	0.03, *0.091* [−0.00;0.06] (0.40)
	Generic	40.67 (12.84)	28.08 (13.96)	24.56 (13.9)		
SCAS adolescent	Disorder-specific	47.11 (16.24)	33.75 (14.46)	29.4 (14.47)	− 0.12, *0.000* [−0.14;−0.1.0] (1.83)	0.04, *0.006* [0.01;0.07] (0.45)
	Generic	55.4 (16.41)	35.57 (17.59)	33.42 (15.25)		
SCAS mother	Disorder-specific	39.0 (16.59)	25.12 (11.14)	24.08 (12.97)	− 0.07, *0.000* [−0.09;−0.05] (1.18)	0.00,.*818* [−0.03;0.03] (0.04)
	Generic	43.93 (13.5)	33.42 (15.05)	30.03 (16.55)		
SCAS father	Disorder-specific	34.81 (14.93)	27.24 (13.92)	21.1 (13.03)	− 0.06, *0.000* [−0.09;−0.03=] (1.03)	0.00, *0.860* [−0.04;0.05] (0.04)
	Generic	40.6 (17.57)	31.45 (19.11)	28.41 (16.28)		
CALIS adolescent	Disorder-specific	16.46 (6.5)	13.18 (8.1)	11.48 (6.86)	− 0.03, *0.000* [−0.04;−0.02] (1.50)	0.01,.*105* [−0.00;0.03] (0.38)
	Generic	19.48 (6.57)	14.0 (6.73)	13.3 (7.2)		
CALIS mother	Disorder-specific	34.36 (10.72)	25.21 (12.04)	22.05 (13.09)	− 0.07, *0.000* [−0.09;−0.05] (1.54)	0.01, *0.596* [−0.02;0.03] (0.12)
	Generic	35.83 (10.89)	29.03 (11.57)	22.26 (13.54)		
CALIS father	Disorder-specific	28.24 (11.79)	23.65 (12.47)	17.33 (12.03)	− 0.04, *0.000* [−0.06;−0.02] (0.66)	− 0.00, *0.775* [−0.03;0.03] (0.05)
	Generic	29.4 (11.73)	24.39 (13.49)	20.94 (13.54)		
S-MFQ adolescent	Disorder-specific	11.13 (6.15)	7.58 (6.23)	6.7 (6.46)	− 0.03, *0.000* [−0.04; − 0.02] (1.21)	0.01, *0.130* [−0.00;0.03] (0.35)
	Generic	14.33 (6.06)	9.95 (7.01)	8.06 (6.75)		
S-MFQ mother	Disorder-specific	10.51 (6.42)	6.36 (5.55)	5.71 (5.3)	− 0.03, *0.000* [−0.04;−0.02] (1.12)	0.01, *0.224* [−0.01;0.02] (0.19)
	Generic	11.34 (5.37)	7.79 (6.42)	4.37 (3.99)		
S-MFQ father	Disorder-specific	8.36 (5.59)	5.78 (5.69)	3.63 (4.21)	− 0.02,.*000* [−0.03;−0.01] (0.69)	− 0.00, *0.808* [−0.02;0.01] (0.04)
	Generic	9.06 (6.22)	6.85 (6.62)	5.85 (5.52)		
DASS mother	Disorder-specific	16.96 (16.28)	12.62 (12.36)	8.71 (10.71)	− 0.03,.*024* [−0.05;−0.00] (0.57)	− 0.02, *0.340* [−0.05;0.02] (0.23)
	Generic	13.1 (12.57)	13.13 (14.17)	6.77 (7.64)		
DASS father	Disorder-specific	11.12 (13.83)	10.69 (20.81)	6.13 (8.96)	− 0.02, *0.325* [−0.06;0.02] (0.17)	0.00, *0.994* [−0.05;0.05] (0.00)
	Generic	13.66 (13.96)	11.61 (22.44)	9.03 (13.91)		
CATS adolescent	Disorder-specific	28.22 (16.7)	19.28 (17.41)	15.97 (16.44)	− 0.08, *0.000* [−0.11;−0.05] (1.22)	0.03, *0.195* [−0.01;0.07] (0.31)
	Generic	36.4 (20.46)	22.62 (18.86)	20.52 (19.22)		
SAFE adolescent	Disorder-specific	85.57 (17.09)	70.78 (17.07)	65.39 (18.76)	− 0.13, *0.000* [−0.17;−0.09] (1.49)	0.048 *0.153* [−0.01;0.09] (0.32)
	Generic	92.6 (19.0)	72.11 (21.65)	67.66 (20.7)		

*SPIN* Social Phobia Inventory, *SCAS* Spence Children’s Anxiety Scale, *CALIS total* Child Anxiety Life Interference, *S-MFQ* Short version of the Mood and Feelings Questionnaire, *CATS* Children’s Automatic Thoughts Scale, *SAFE* Subtle Avoidance Frequency Examination. Note that for the primary outcome measures (SPIN, SCAS), we applied an adjusted significance level (α *< 0.005)*

At post-treatment, 11 (25%) adolescents in disorder-specific group CBT and 7 (18.4%) in generic group CBT were free of their social anxiety diagnosis. By the 3-month follow-up, remission rates increased to 22 (55%) in the disorder-specific CBT and 17 (48.6%) in the generic CBT. This difference was non-significant, OR = 1.01, *α* = 0.705. However, regardless of treatment condition, over time the remission from social anxiety diagnosis was significant for both treatment conditions, OR = 0.92, *α* = 0.003.

The Social Phobia Inventory (SPIN) also revealed a non-significant difference between the two treatment conditions regarding reduction in social anxiety symptoms, β = 0.03, *α* = 0.091, *d* = 0.40. However, SPIN revealed a significant reduction in social anxiety symptoms from baseline to 3-month follow-up for both treatment conditions, β = − 0.09, *α* = 0.000, *d* = 1.81.

Spence Children’s Anxiety Scale (SCAS) similarly demonstrated a significant reduction in overall anxiety symptoms for both treatment conditions reported by adolescents, mothers, and fathers. No significant differences were found between the treatment conditions when looking at the mothers’ and fathers’ reports. But adolescents in the generic CBT reported greater improvement in overall anxiety symptoms, β = 0.04, *α* = 0.006, *d* = 0.45. However, when applying the adjusted significance level (*α* < 0.005), the difference for the adolescents’ reports on overall anxiety symptoms was no longer significant.

### Secondary Outcome Measures

There was no significant time-by-group interaction effect for any secondary outcome measure. However, regardless of treatment conditions, all secondary outcome measures showed a significant reduction over time, with effect sizes ranging from *d =* 0.57–1.54.57.54, equivalent to medium to large effects. The only exception to this was the fathers’ reports on DASS, measuring fathers’ anxiety, stress, and depression levels, which, regardless of treatment conditions, did not reach a significant reduction over time, β = − 0.02, *p* =.325, *d* = 0.17.

#### Treatment Satisfaction

Overall, the participants were satisfied with the treatment. In the disorder-specific group CBT adolescents had a mean of 10.3 (2.4 SD) out of a maximum score of 14, the mothers had a mean of 18.0 (2.3 SD) out of a maximum of 20, and the fathers had a mean of 17.2 (3.9 SD) out of a maximum of 20. In the generic group CBT adolescents had a mean of 10.2 (2.9 SD) out of a maximum score of 14, the mothers had a mean of 18.1 (2.0 SD) out of a maximum of 20, and the fathers had a mean of 17.5 (3.5 SD) out of a maximum of 20. There were no significant differences in treatment satisfaction between the treatment conditions based on reports from adolescents (t(75) = 0.01, *p* =.991, *d* = 0.00), mothers (t(78) = − 0.28, *p* =.392, *d* = 0.06) or fathers (t(66) = − 0.32, *p* =.376, *d* = 0.08).

#### Negative Effects

In the disorder-specific group CBT 40 adolescents completed the questionnaire post-treatment. Of those, a total of 26 (65.0%) adolescents reported negative effects following treatment due to either treatment or treatment and other circumstances, with an average impact of 1.7 (1.0 SD). A total of 25 (59.5%) mothers reported negative effects with an average impact of 1.7 (0.7 SD), and 20 (57.1%) fathers with an average impact of 2.3 (1.0 SD).

For the generic group CBT 37 adolescents completed the NEQ questionnaire post-treatment. Of those, a total of 23 (62.1%) adolescents reported negative effects following treatment due to either treatment or treatment and other circumstances, with an average impact of 1.7 (0.9 SD). A total of 20 (54.1%) mothers reported negative effects with an average impact of 2.1 (1.0 SD), and a total of 12 (36.4%) fathers with an average impact of 2.2 (1.2 SD).

For more information on the specific items see Table 4 and Appendix A.

**Table 4:** Negative effects reported by the adolescents

Item		Frequency, *n* (%)	Negative impact, M (SD)^a^
1. I Had more problems with my sleep	Disorder-specific	5 (12.5)	1.8 (0.8)
	Generic	2 (5.4)	2.5 (0.7)
2. Felt like I was more stressed	Disorder-specific	11 (27.5)	1.7 (0.9)
	Generic	9 (24.3)	2.0 (1.2)
3. I experienced more anxiety	Disorder-specific	10 (25.0)	2.5 (1.1)
	Generic	11 (29.7)	1.9 (1.4)
4. I felt more worried	Disorder-specific	5 (12.5)	1.8 (1.3)
	Generic	5 (13.5)	2.0 (1.2)
5. I experienced more hopelessness	Disorder-specific	8 (20.0)	2.1 (1.1)
	Generic	2 (5.4)	3.5 (0.7)
6. I experienced more unpleasant feelings	Disorder-specific	10 (25.0)	1.6 (1.0)
	Generic	7 (18.9)	1.1 (1.2)
7. I felt like the issue I was looking for help with got worse	Disorder-specific	2 (5.0)	3.5 (0.7)
	Generic	2 (5.4)	3.0 (0.0)
8. Unpleasant memories resurfaced	Disorder-specific	9 (22.5)	1.9 (1.3)
	Generic	7 (18.9)	1.6 (1.1)
9. I became afraid that other people would find out about my treatment	Disorder-specific	3 (7.5)	1.7 (1.2)
	Generic	8 (21.6)	1.8 (0.7)
10. I got thoughts that it would be better if I did not exist anymore and I should take my own life	Disorder-specific	1 (2.5)	2.0 (0.0)
	Generic	2 (5.4)	2.5 (2.1)
11. I started feeling ashamed because I was having treatment	Disorder-specific	1 (2.5)	3.0 (0.0)
	Generic	2 (5.4)	1.0 (0.0)
12. I stopped thinking that things could get better	Disorder-specific	6 (15.0)	2.8 (0.8)
	Generic	5 (13.5)	2.8 (1.3)
13. I started thinking that the issue I was seeking help for could not be made any better	Disorder-specific	7 (17.5)	2.6 (1.1)
	Generic	6 (16.2)	2.5 (1.0)
14. I think that I have developed a dependency on my treatment	Disorder-specific	5 (12.5)	0.8 (1.3)
	Generic	0	0
15. I did not always understand my treatment	Disorder-specific	10 (25.0)	1.4 (0.8)
	Generic	3 (8.1)	2.0 (1.7)
16. I did not always understand my therapist	Disorder-specific	3 (7.5)	1.0 (0.0)
	Generic	0	0
17. I did not have confidence in my treatment	Disorder-specific	7 (17.5)	1.3 (0.5)
	Generic	5 (13.5)	1.4 (1.7)
18. I felt that the treatment did not produce any results	Disorder-specific	4 (10.0)	2.3 (0.5)
	Generic	6 (16.2)	2.8 (1.2)
19. I felt that my expectations for the therapist were not fulfilled	Disorder-specific	0	0
	Generic	1 (2.7)	2.0 (0.0)
20. I felt that the treatment was not motivating	Disorder-specific	3 (7.5)	1.3 (0.6)
	Generic	3 (8.1)	2.0 (1.0)

Note. The rating reflects the negative effects caused by the treatment, or by the treatment *and* other circumstances, *n* = 196 (54.4%). Thereby not showing negative effects only caused by other circumstances. ^a^The intensity of how negatively it affected was rated on a 5-point Likert scale ranging from 0–4. 0 = Not at all, 1 = Slightly, 2 = Moderately, 3 = Very, 4 = Extremely

We did not directly compare our results on negative effects with those of other studies because participants in our study were allowed to attribute negative effects to both “the treatment I received” and “other circumstances.” Consequently, many respondents attributed the negative effects to both the treatment and other circumstances. In contrast, participants in most other studies are typically required to attribute negative effects to either “the treatment I received” or “other circumstances,” but not both. This methodological difference makes it difficult to directly compare our findings with those of other studies.

## Discussion

To the best of our knowledge, the current study was the first randomized controlled trial to compare a disorder-specific CBT in a group format, specifically enhanced for adolescents with social anxiety disorder to a generic CBT in a group format, designed for all types of anxiety disorders, in adolescents with primary social anxiety disorder.

We hypothesized that both treatment conditions would exhibit a reduction in social anxiety symptoms, anticipating that the disorder-specific group CBT would demonstrate a greater reduction. Contrary to our expectations, the results indicated comparable results with non-to-small non-significant differences between the two treatment conditions over time on the outcome measures. The only exception was adolescents’ self-reported overall anxiety levels, measured by the Spence Children’s Anxiety Scale (SCAS), where a medium and significant interaction effect for time and treatment conditions was found favoring the generic group CBT. However, when applying the adjusted significance level, the difference in the adolescents’ self-reported overall anxiety level was no longer statistically significant.

Anyhow, a significant and large improvement over time was found on all outcome measures regardless of treatment conditions. The only exception to this was the fathers’ self-reported stress, anxiety, and depression levels measured by the Depression Anxiety Stress Scale (DASS), which revealed a small non-significant reduction over time in both treatment conditions.

The results of the current study aligned with findings from the study by Rapee et al. (2023), which examined identical treatment conditions but in an individual treatment format. In concordance with our findings, their study found no significant differences between the two treatment conditions across any outcome measure, while both groups showed substantial reduction across all outcomes over time. However, at their 6-month follow-up, they observed a trend (*p* =.08) favoring the disorder-specific CBT where 68.8% of participants were free from their social anxiety disorder compared to 51.2% in the generic CBT. Despite having the same treatment conditions and method, there were notable differences between the study conducted by Rapee et al. (2023) and our study. Most significantly, their treatment was administered in an individual format, in contrast to our group format. Additionally, their study included a broader age range (7–17 years old), did not require social anxiety disorder to be the primary disorder, and excluded adolescents with ‘frequent school nonattendance’. In contrast, our study focused exclusively on adolescents aged 12–17, with social anxiety disorder as their primary disorder, and frequent school absenteeism was not an exclusion criterion. This may indicate a more complex sample in the current study, particularly due to the inclusion of participants with frequent school absenteeism, older sample, and primary social anxiety disorder. These factors could potentially make recovery more challenging in the current sample. Despite these differences, the alignment of results between both studies, which used the same treatment conditions and method, suggests the reliability of the findings.

Two other randomized controlled trials investigated disorder-specific CBT against a generic CBT for social anxiety disorder, with results comparable to our findings. Spence et al. (2017) conducted a randomized controlled trial and did not find any significant differences between the individual disorder-specific CBT and the individual generic CBT for children and adolescents aged 8–17 years with a primary social anxiety disorder. However, it is worth noting that both treatment conditions were delivered online. Ingul et al. (2014) also conducted a randomized controlled trial for adolescents aged 13–16 with a primary social anxiety disorder. They found that the disorder-specific individual CBT was significantly better on the self-reported measures but found no significant differences in the semi-structured diagnostic interview. However, they compared an individual disorder-specific CBT to a group generic CBT, which made the results more complex to interpret, as the results could stem from variations in format, treatment methods, or a combination of both factors.

Since the results in the current study aligned well with the previous results, an important question is why we do not see any notable differences between the disorder-specific CBT and the generic CBT?

One possible explanation could be that more time is required for change to occur. In the current study, both treatment conditions showed significant reduction over time. The same tendency was observed in the study by Rapee et al. (2023), and further on they found a trend in remission from social anxiety disorder toward disorder-specific CBT at their 6-month follow-up. Hence, it is possible, that while disorder-specific CBT may not demonstrate immediate advantages, the additional techniques may take longer to be learned and effectively applied in real-life situations. With extended follow-up periods, differences between treatment conditions may become more apparent, suggesting potential long-term benefits of disorder-specific CBT.

Another possible explanation for the observed lack of difference between the disorder-specific CBT and the generic CBT could be that the presence of shared standard CBT techniques in both conditions contributes significantly to the overall effect. There were several shared treatment techniques, such as psychoeducation about social anxiety, exposure, in-vivo exposure, and homework. Exposure and in-vivo exposure have been recognized as central techniques for achieving a positive effect in CBT (Kazantzis et al., 2018; Whiteside et al., 2020). Adolescents in the generic group CBT had fewer treatment techniques to learn, which provided more time for learning exposure and practicing in-vivo exposure, which was considered central to obtaining an effect in CBT (Whiteside et al., 2020).

Alternatively, another possible explanation for the observed lack of difference is that the disorder-specific treatment techniques were the most important but required more time during treatment sessions to be learned and implemented in the adolescents’ lives. In disorder-specific CBT for adults, based on the Clark and Wells (1995) model, standard CBT techniques, such as psychoeducation, are often not included. Instead, treatment focuses solely on the disorder-specific techniques specially developed to target the maintenance processes related to social anxiety disorder, such as safety behavior, self-focused attention, and post-event processing (Leigh, 2023; Mayo-Wilson et al., 2014). In the current study the disorder-specific techniques, such as post-event processing, safety behavior, and self-focused attention, were introduced in sessions 3, 6, and 6, respectively (see Table 1). Compared to the adult disorder-specific CBT, based on Clark and Wells (1995), this was fairly late. Therefore, it is suggestable that if the disorder-specific CBT techniques had had more focus and time in sessions from the beginning of the treatment they might have shown better effects.

Nonetheless, it is important to acknowledge that we do not have evidence confirming whether the participants actually acquired the treatment techniques introduced during the sessions. This means that while the discussion points raised are relevant, we did not specifically assess whether the participants learned and applied the skills.

However, regardless of treatment condition, we found a significant and medium to large effect size on nearly all outcome measures in the current study. This indicates that the group format for treating adolescents with social anxiety disorder might be a feasible approach. When comparing the results from the current study to the broader literature on treatment of social anxiety disorder, our findings aligned. Two meta-analyses reported remission rates from social anxiety disorder between 21 and 62% (Evans et al., 2021) and 14–69% (Yang et al., 2019). In comparison to the remission rates from the semi-structured diagnostic interview in the current study, our results fell within the lower end at post-treatment. However, the reduction over time in the self-reported outcome measures was comparable to existing literature, demonstrating significant medium to large effect sizes. Combined with the high treatment satisfaction and low dropout rate (4.7%) in the current study it supports the suggestion that the group format with participants exclusively diagnosed with primary social anxiety disorder was a feasible and well-received approach for adolescents with social anxiety disorder.

Finally, it seems crucial to understand why social anxiety disorder among adolescents is associated with fewer treatment outcomes compared to other anxiety disorders. Even though it is a well-known issue, the reasons for these poorer outcomes in adolescents with social anxiety disorder are not yet clear. One possible explanation may be the cognitive development in adolescence, where self-consciousness increases, leading to heightened awareness of the self as a social object (Rankin et al., 2004). The increase in self-consciousness has been found related to social anxiety disorder and its maintenance (Leigh & Clark, 2018; Tillfors & Van Zalk, 2015). Additionally, fluid self-concepts and increased sensitivity to peer opinions and peer rejection make adolescents more susceptible to negative aspects of peer interactions (Leigh & Clark, 2018; Rapee et al., 2019b). This vulnerability, combined with the cognitive aspects of heightened self-consciousness, may contribute to the complexity of treating social anxiety disorder in adolescents. Moreover, social anxiety disorder is believed to be closely connected to personality and temperament, which can plausibly make improvements slower or more challenging. In particular, behavioral inhibition is associated with the development and maintenance of social anxiety disorder (Rapee, 2002; Spence & Rapee, 2016). Behavioral inhibition is characterized by behavioral withdrawal and avoidance. Some of the main characteristics of behaviorally inhibited temperament include limited eye contact, lack of verbal utterances, and avoidance, which are also symptoms of social anxiety disorder and its maintenance (Spence & Rapee, 2016). Altogether, the above factors may make social anxiety disorder more difficult to treat or at least require a longer treatment duration, potentially explaining some of the weaker effects of CBT treatments compared to its effectiveness for other anxiety disorders.

### Strength and Limitations

The current study has several strengths, including standardized assessments, self-reported outcomes from adolescents, and ratings from both parents and clinicians. Additionally, there was an active comparator and low attrition rates.

However, there were some limitations worth mentioning. Firstly, both treatment conditions became somewhat disorder-specific, as all participants suffered from social anxiety disorder. As a result, all psychoeducation, exposure, and all examples were focused solely on social anxiety disorder. Even the generic group CBT was focused exclusively on social anxiety disorder, making it somewhat disorder-specific as well. Even though there were some advantages of having the same therapists deliver both treatment conditions, it also came with some limitations. Related to this, there were 13 fidelity breaches, where therapists in the generic group CBT used treatment techniques intended exclusively for the disorder-specific group CBT. These breaches may have made the generic group CBT more disorder-specific, and altogether, this could make it more challenging to identify differences. Secondly, there was no long-term follow-up, which could have revealed whether the reduction in symptoms would be maintained or if the differences between treatment conditions would be more pronounced, as seen in the study by Rapee et al. (2023). Moreover, the initial power analysis indicated that a total of 94 adolescents (47 in each treatment condition) were needed to obtain sufficient power. With a final sample size of 90, the study was slightly underpowered. In addition to this, based on results from published studies (e.g. Rapee et al., 2023; Spence et al., 2017) after the power analysis was conducted, these findings may indicate that the estimated effect size was relatively high, suggesting that the study could have benefited from a larger sample size. Additionally, 73 (81.1%) of the sample identified as girls, leaving only 17 boys (18.9%) in the sample. Of these, 13 were included in the disorder-specific group CBT, whereas only 4 were allocated to the generic group CBT, resulting in a significant difference in gender distribution between treatment conditions. This pattern is consistent with previous studies, which have also found girls to be overrepresented in treatment studies of social anxiety disorder in similar age groups (e.g., Alves et al., 2022; Esbjørn et al., 2010). Although the total number of boys was relatively small, this significant difference should be taken into account when interpreting the findings, as gender may be associated with differences in symptoms and treatment response.

Finally, although ADIS-IV C/P is considered the golden standard diagnostic interview, it seems inevitable that each center has its own standards for Clinical Severity Rating, which can make it challenging to compare the ADIS-IV C/P results directly with those from other studies. In contrast, standardized questionnaires provide a more straightforward basis for comparison. Despite that, ADIS-IV C/P remains a valuable tool for evaluating anxiety disorders.

## Conclusion

The outcomes for adolescents with a social anxiety disorder are still lower than for other anxiety disorders (Evans et al., 2021). Therefore, it seems crucial to improve treatments for social anxiety disorder and identify the most effective methods.

The current study was the first to compare a disorder-specific group CBT to a generic group CBT for adolescents suffering from social anxiety disorder. Contrary to our initial hypothesis, we found no significant differences between the two treatment conditions over time on all outcome measures after adjusting for multiple comparisons. However, we did observe a statistically significant improvement over time regardless of the treatment conditions. At the 3-month follow-up, approximately 50% of the adolescents in both treatment conditions achieved remission from social anxiety disorder. Additionally, we observed a significant reduction in social anxiety symptoms, overall anxiety symptoms, life interferences, and depression levels. Moreover, both treatment groups displayed high rates of treatment satisfaction and low attrition rates. These findings imply that adolescents with social anxiety disorder can be successfully treated in a group format together with others with primary social anxiety disorder. Nevertheless, future research should explore ways to optimize the treatment effect for adolescents with social anxiety disorder. It may be beneficial to investigate mediating measures that can elucidate the underlying mechanism of treatment effects, potentially contributing to the understanding of which treatment techniques to move forward with. Additionally, investigating moderators or predictors could help identify subgroups that may benefit more from CBT treatments.

## Supplementary Information

Below is the link to the electronic supplementary material.

Supplementary Material 1 (DOCX 24.5 KB)

## Data Availability

The dataset will be anonymized and made available on request.
