# Supplementary material for: Comparison of Disorder-Specific Group CBT and Generic Group CBT in Treating Adolescents with Social Anxiety Disorder: A Randomized Controlled Trial
Source: Res Child Adolesc Psychopathol. 2026 Feb 6;54(1):27. doi: 10.1007/s10802-025-01412-z (PMC12881119; doi:10.1007/s10802-025-01412-z)
Supplement: Supplementary file 1 — Supplementary Material 1 (DOCX 24.5 KB) [file 10802_2025_1412_MOESM1_ESM.docx]

## **Supplementary**

**Appendix:** Negative effects reported by the mothers and fathers

| **Item** |  | **Frequency, n (%)** | **Negative impact,  M (SD)^a^** |
| --- | --- | --- | --- |
| *Mothers’ reports* |  |  |  |
| 1. My child had more problems with sleep | Disorder-specific | 0 | 0 |
|  | Generic | 1 (2.7) | 3.0 (0.0) |
| 2. My child felt more stressed | Disorder-specific | 11 (26.2) | 1.9 (0.7) |
|  | Generic | 14 (37.8) | 1.6 (1.0) |
| 3. My child experienced more anxiety | Disorder-specific | 9 (21.4) | 2.0 (0.7) |
|  | Generic | 12 (32.4) | 1.9 (1.0) |
| 4. My child felt more worried | Disorder-specific | 2 (4.8) | 1.0 (1.4) |
|  | Generic | 2 (5.4) | 1.5 (0.7) |
| 5. My child experienced more hopelessness | Disorder-specific | 3 (7.1) | 2.0 (1.7) |
|  | Generic | 6 (16.2) | 2.5 (1.0) |
| 6. My child experienced more unpleasant feelings | Disorder-specific | 9 (21.4) | 2.1 (0.8) |
|  | Generic | 10 (27.0) | 1.6 (0.8) |
| 7. My child felt like the issue he/she was looking for help with got worse | Disorder-specific | 5 (11.9) | 2.2 (1.1) |
|  | Generic | 3 (8.1) | 2.3 (1.5) |
| 8. My child’s unpleasant memories resurfaced | Disorder-specific | 8 (19.0) | 1.3 (0.5) |
|  | Generic | 1 (2.7) | 1.0 (0.0) |
| 9. My child became afraid that other people would find out about his/her treatment | Disorder-specific | 5 (11.9) | 1.8 (0.8) |
|  | Generic | 5 (13.5) | 2.0 (0.7) |
| 10. My child had thoughts that it would be better if he/she did not exist anymore and that he/she should take his/her own life | Disorder-specific | 0 | 0 |
|  | Generic | 1 (2.7) | 4.0 (0.0) |
| 11. My child started feeling ashamed because he/she was having treatment | Disorder-specific | 2 (4.8) | 1.0 (0.0) |
|  | Generic | 2 (5.4) | 2.0 (1.4) |
| 12. My child stopped thinking that things could get better | Disorder-specific | 7 (16.7) | 2.2 (0.8) |
|  | Generic | 4 (10.8) | 2.5 (1.0) |
| 13. My child started thinking that the issue he/she was seeking help for could not be made any better | Disorder-specific | 8 (19.0) | 2.0 (0.8) |
|  | Generic | 9 (24.3) | 2.1 (1.2) |
| 14. My child thinks that he/she developed a dependency on the treatment | Disorder-specific | 0 | 0 |
|  | Generic | 2 (5.4) | 1.5 (0.7) |
| 15. My child did not always understand the treatment | Disorder-specific | 13 (31.0) | 1.5 (0.9) |
|  | Generic | 9 (24.3) | 1.9 (0.8) |
| 16. My child did not always understand the therapist | Disorder-specific | 4 (9.5) | 1.5 (0.6) |
|  | Generic | 2 (5.4) | 1. (0.0) |
| 17. My child did not have confidence in the treatment | Disorder-specific | 7 (16.7) | 1.7 (0.8) |
|  | Generic | 8 (21.6) | 2.1 (1.0) |
| 18. My child felt that the treatment did not produce any results | Disorder-specific | 8 (19.0) | 1.6 (1.2) |
|  | Generic | 6 (16.2) | 2.1 (1.1) |
| 19. My child felt that his/her expectations for the therapist were not fulfilled | Disorder-specific | 1 (2.4) | 2.0 (0.0) |
|  | Generic | 3 (8.1) | 2.0 (1.0) |
| 20. My child felt that the treatment was not motivating | Disorder-specific | 4 (9.5) | 2.0 (0.8) |
|  | Generic | 5 (13.5) | 2.6 (0.5) |
| *Fathers’ reports* |  |  |  |
| 1. My child had more problems with sleep | Disorder-specific | 1 (2.9) | 4.0 (0.0) |
|  | Generic | 0 | 0 |
| 2. My child felt more stressed | Disorder-specific | 13 (37.1) | 2.0 (1.2) |
|  | Generic | 8 (24.2) | 2.2 (0.8) |
| 3. My child experienced more anxiety | Disorder-specific | 8 (22.9) | 2.1 (1.1) |
|  | Generic | 4 (12.1) | 2.8 (1.0) |
| 4. My child felt more worried | Disorder-specific | 3 (8.6) | 2.7 (1.2) |
|  | Generic | 2 (6.1) | 3.0 (1.4) |
| 5. My child experienced more hopelessness | Disorder-specific | 4 (11.4) | 2.3 (1.3) |
|  | Generic | 5 (15.2) | 2.2 (1.3) |
| 6. My child experienced more unpleasant feelings | Disorder-specific | 6 (17.1) | 2.0 (1.4) |
|  | Generic | 4 (12.1) | 2.3 (1.3) |
| 7. My child felt like the issue he/she was looking for help with got worse | Disorder-specific | 7 (20.0) | 2.1 (1.1) |
|  | Generic | 6 (18.2) | 2.2 (1.2) |
| 8. My child’s unpleasant memories resurfaced | Disorder-specific | 7 (20.0) | 1.7 (1.1) |
|  | Generic | 4 (12.1) | 2.3 (1.7) |
| 9. My child became afraid that other people would find out about his/her treatment | Disorder-specific | 7 (20.0) | 1.9 (1.1) |
|  | Generic | 5 (15.2) | 2.0 (1.2) |
| 10. My child had thoughts that it would be better if he/she did not exist anymore and that he/she should take his/her own life | Disorder-specific | 2 (5.7) | 3.0 (0.0) |
|  | Generic | 1 (3.0) | 4.0 (0.0) |
| 11. My child started feeling ashamed because he/she was having treatment | Disorder-specific | 3 (8.6) | 2.3 (1.5) |
|  | Generic | 2 (6.1) | 2.5 (2.1) |
| 12. My child stopped thinking that things could get better | Disorder-specific | 4 (11.4) | 2.0 (1.4) |
|  | Generic | 5 (15.2) | 2.2 (1.3) |
| 13. My child started thinking that the issue he/she was seeking help for could not be made any better | Disorder-specific | 5 (14.3) | 1.2 (1.2) |
|  | Generic | 5 (15.2) | 2.0 (1.2) |
| 14. My child thinks that he/she developed a dependency on the treatment | Disorder-specific | 1 (2.9) | 4.0 (2.1) |
|  | Generic | 2 (6.1) | 2.5 (0.0) |
| 15. My child did not always understand the treatment | Disorder-specific | 7 (20.0) | 2.7 (1.2) |
|  | Generic | 3 (9.1) | 2.0 (1.7) |
| 16. My child did not always understand the therapist | Disorder-specific | 3 (8.6) | 2.5 (2.1) |
|  | Generic | 2 (6.1) | 0 |
| 17. My child did not have confidence in the treatment | Disorder-specific | 3 (8.6) | 2.0 (1.3) |
|  | Generic | 7 (21.2) | 1.4 (1.7) |
| 18. My child felt that the treatment did not produce any results | Disorder-specific | 3 (8.6) | 2.3 (1.5) |
|  | Generic | 4 (12.1) | 2.8 (1.2) |
| 19. My child felt that his/her expectations for the therapist were not fulfilled | Disorder-specific | 1 (2.9) | 2.0 (1.2) |
|  | Generic | 5 (15.2) | 2.0 (0.0) |
| 20. My child felt that the treatment was not motivating | Disorder-specific | 0 | 0 |
|  | Generic | 2 (6.1) | 3.0 (1.4) |

**Note.** The rating reflects the negative effects caused by the treatment, or by the treatment *and* other circumstances, for mothers: n=211 (67.4%), for fathers: n=164 (68.9%). Thereby not showing negative effects caused only by other circumstances. ^a^The intensity of how negatively it affected was rated on a 5-point Likert scale ranging from 0-4. 0 = Not at all, 1 = Slightly, 2 = Moderately, 3 = Very, 4 = Extremely.
